# Recent advances in pathogenesis, assessment, and treatment of atherosclerosis

**DOI:** 10.12688/f1000research.8459.1

**Published:** 2016-07-28

**Authors:** J. David Spence

**Affiliations:** 1 Stroke Prevention & Atherosclerosis Research Centre, Robarts Research Institute, Western University, London, ON, Canada

**Keywords:** atherosclerosis, carotid plaque, carnitine, LDL, cholesterol, Transcranial Doppler embolus detection

## Abstract

In recent years, there have been a number of advances in the pathogenesis and treatment of atherosclerosis and in assessing prognosis in carotid atherosclerosis. Risk stratification to improve vascular prevention by identifying patients most likely to benefit from intensive therapy is much improved by measuring carotid plaque burden. In patients with asymptomatic carotid stenosis, a number of modalities can be used to identify the 10-15% who could benefit from endarterectomy or stenting. Transcranial Doppler embolus detection, echolucency and ulceration on 3D ultrasound, intraplaque hemorrhage on magnetic resonance imaging (MRI), and reduced cerebrovascular reserve are useful already; new approaches including plaque texture on ultrasound and imaging of plaque inflammation and early calcification on positron emission tomography/computed tomography (PET/CT) are in development. The discovery that the intestinal microbiome produces vasculotoxic metabolites from dietary constituents such as carnitine in meat (particularly red meat) and phosphatidylcholine from egg yolk and other sources has revolutionized nutritional aspects of vascular prevention. Because many of these vasculotoxic metabolites are removed by the kidney, it is particularly important in patients with renal failure to limit their intake of red meat and egg yolk. A new approach to lowering low-density lipoprotein (LDL) cholesterol by blocking the action of an enzyme that destroys LDL receptors promises to revolutionize vascular prevention once less costly treatments are developed, and a new approach to vascular prevention—“treating arteries instead of risk factors”—shows promise but requires randomized trials. These advances all promise to help in the quest to prevent strokes in high-risk patients.

## Introduction

In recent years, there have been important advances in the pathogenesis and treatment of atherosclerosis and in assessing prognosis in carotid atherosclerosis. The effect of the intestinal microbiome on atherosclerosis has revolutionized thinking about diet ^1^ and about the role of renal failure in increasing cardiovascular risk. In the past, routine treatment with usual therapy for atherosclerosis has reduced cardiovascular risk in most trials by only ~9–30% ^2^, resulting in a residual risk of 70–80% ^3– 6^. Recent advances in lipid-lowering therapy, based on a novel mechanism based on blocking the effects of an enzyme that destroys receptors for low-density lipoprotein (LDL) cholesterol, make it possible to lower LDL to a greater degree and in more patients than was previously possible ^7^. Lifelong reduction of LDL resulting from a hereditary cause of low levels of LDL results in a reduction of coronary risk by ~95% ^8, 9^, and a new approach to therapy based on “treating arteries instead of treating risk factors” ^10^ reduced the very high risk in patients with asymptomatic carotid stenosis by more than 80% ^11^. Most patients (~90%) with asymptomatic carotid stenosis would be better treated by intensive medical therapy than by carotid endarterectomy (CEA) or carotid artery stenting (CAS) ^12^. Methods to identify the few (10–15%) who could benefit from intervention are being developed ^13^. In this review, I focus on advances in the understanding of the role of the intestinal microbiome and renal impairment on atherosclerosis, measurement of carotid plaque burden, carotid ulceration and ulcer volume, plaque texture, and detection of microemboli by transcranial Doppler (TCD).

## Advances in pathogenesis of atherosclerosis

Atherosclerosis may be thought of as a response to injury ^14– 16^, related to flow disturbances that injure the endothelium ^17^, followed by adhesion of platelets, penetration of macrophages into the subendothelium, inflammation ^18^, oxidative stress, LDL oxidation, and proliferation of smooth muscle cells, similar to a scar in the artery wall. Risk factors identified in the Framingham Heart Study included hypertension, smoking, elevated LDL, diabetes, and left ventricular hypertrophy (essentially reflecting the integral of blood pressure over recent years). The importance of diet has been largely unappreciated, perhaps because lowering of fasting LDL with drugs such as statins has seemingly countered any effect of diet on fasting lipids. However, this misplaced focus on fasting lipids misses the key effects of diet, which occur during the post-prandial state ^19^. A high-fat/high-cholesterol meal increases arterial inflammation ^20, 21^ and oxidative stress, and impairs endothelial function ^22^, for several hours. Most of the day is spent in the post-prandial state, so diet is much more important than would be predicted by fasting LDL.

Several studies have shown that healthy lifestyle choices (not smoking, moderate alcohol intake, regular exercise, consuming a healthy diet, and maintaining a healthy weight) markedly reduce the risk of stroke. In the US Health Professionals Study and the Nurses’ Health study, persons who adopted all of these choices had an 80% reduction of stroke ^23^; Swedish men with coronary artery disease who did so also had an 80% reduction of recurrent myocardial infarction ^24^. The Cretan Mediterranean diet reduced cardiovascular events by 70% in secondary prevention ^25^ and reduced stroke by nearly half in high-risk primary prevention ^26^. It has been clear for many years that dietary cholesterol increases coronary risk ^27^. Now there is a completely new window opening on the relationship between diet and atherosclerosis, relating to the intestinal microbiome ^1^.

## Intestinal microbiome and diet

**Figure 1.**
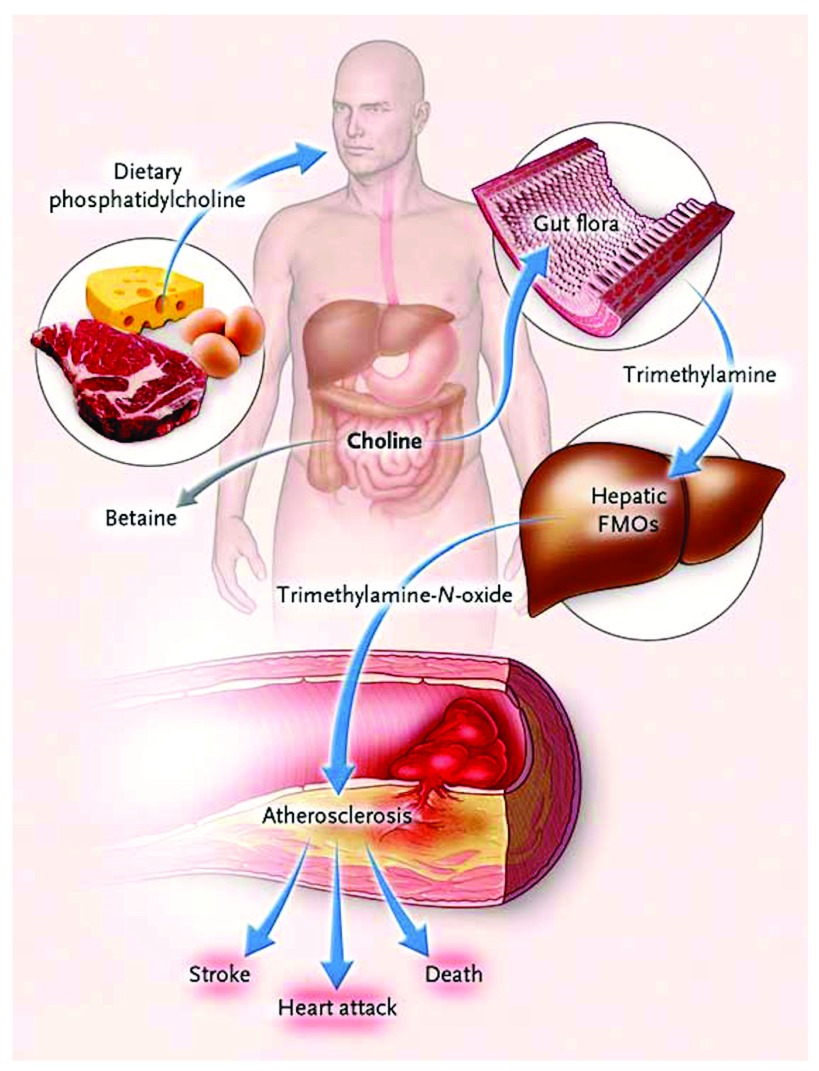
Pathways linking dietary phosphatidylcholine, intestinal microbiota, and incident adverse cardiovascular events. Ingested phosphatidylcholine (lecithin), the major dietary source of total choline, is acted on by intestinal lipases to form a variety of metabolic products, including the choline-containing nutrients glycerophosphocholine, phosphocholine, and choline. Choline-containing nutrients that reach the cecum and large bowel may serve as fuel for intestinal microbiota (gut flora), producing trimethylamine (TMA). TMA is rapidly further oxidized to trimethylamine N-oxide (TMAO) by hepatic flavin-containing monooxygenases (FMOs). TMAO enhances the accumulation of cholesterol in macrophages, the accumulation of foam cells in artery walls, and atherosclerosis, all factors that are associated with an increased risk of heart attack, stroke, and death. Choline can also be oxidized to betaine in both the liver and the kidneys. Dietary betaine can serve as a substrate for bacteria to form TMA and presumably TMAO. Reproduced by permission of the Massachusetts Medical Society from: Tang WHW, Wang Z, Levison BS, Koeth RA, Britt EB, Fu X, Wu Y, Hazen SL: Intestinal Microbial Metabolism of Phosphatidylcholine and Cardiovascular Risk. N Engl J Med 2013, 368(17): 1575–1584.

In recent years, it has become clear that an important interaction between diet and the intestinal microbiome brings into play additional metabolic factors that aggravate atherosclerosis beyond dietary cholesterol. This may help to explain the benefits of the Mediterranean diet. Hazen’s group from the Cleveland Clinic reported that phosphatidylcholine from egg yolk ^28^ and carnitine ^29^ from animal flesh (four times as much in red meat as in fish or chicken) are converted by intestinal bacteria to trimethylamine (the compound that causes uremic breath to smell fishy). Trimethylamine is oxidized in the liver to trimethylamine N-oxide (TMAO) ( Figure 1), which causes atherosclerosis in animal models. In patients referred for coronary angiography, high levels of TMAO following a test dose of two hard-boiled eggs markedly increased risk. Patients in the top quartile of TMAO had a 2.5-fold increase in the 3-year risk of stroke, death, or myocardial infarction ^30^.

A key issue is that vegans who consumed L-carnitine did not produce TMAO because they did not have the intestinal bacteria that produce TMA from carnitine ^29^; this means that the intestinal microbiome is modifiable. A novel approach to treating atherosclerosis would be the eradication of harmful bacteria with antibiotics and recolonization by stool transplantation with beneficial bacteria. This is entirely analogous to the treatment of *Clostridium difficile* infection by “repoopulation” ^31^. Our group is studying that possibility.

## Interaction with renal failure

Patients with renal failure have high levels of total homocysteine (tHcy), but this accounts for only ~20% of the effect of impaired renal function on carotid plaque ^32^. Elevated levels of thiocyanate and asymmetric dimethylarginine (a nitric oxide antagonist) add further to the very high cardiovascular risk ^33, 34^ in renal failure; this is not new. What is recently recognized is that metabolic products of the intestinal microbiome are excreted in the urine, so patients with renal failure also have high levels of TMAO, which accelerate the decline of renal function and increase cardiovascular risk ^35^. Besides TMAO, other metabolic products of the intestinal microbiome that probably contribute to cardiovascular risk in renal failure include indoxyl sulfate, indole-3-acetic acid, p-cresyl sulfate ^32^, and phenylacetylglutamine ^36^. Thus patients at risk of cardiovascular disease should limit their intake of meat and egg yolk not only because of the high cholesterol content but also because of the carnitine in meat (particularly red meat) and the phosphatidylcholine in egg yolk. This is particularly important in patients with renal failure.

## Advances in treatment of atherosclerosis

### Blocking the action of proprotein convertase subtilisin-kexin type 9

Inhibitors of the rate-limiting step in cholesterol synthesis, hydroxymethylglutarate CoA (HMG-CoA) reductase (statins), reduce fasting LDL and reduce cardiovascular events. Those effects are enhanced by combination with ezetimibe, a drug that blocks cholesterol absorption. However, many patients are intolerant of statins. Although there are many myths ^37^ regarding the adverse effects of statins, such as hepatotoxicity, nephrotoxicity, intracerebral hemorrhage, cataracts, and cognitive decline, true causally related adverse effects include myopathy and a slightly increased risk of diabetes. These probably depend mainly on impairment of mitochondrial function ^37^ by depletion of ubiquinone (coenzyme Q10), which is needed for mitochondrial function.

An entirely distinct approach to lowering LDL and reducing cardiovascular events that has recently become available is blocking the action of proprotein convertase subtilisin-kexin type 9 (PCSK9), an enzyme that breaks down LDL receptors. By preventing the breakdown of these receptors, and increasing their number and duration of effect, LDL and cardiovascular events are both lowered by ~50% ^38, 39^. Present approaches to blocking the action of PCSK9—monoclonal antibodies or RNA interference—are very (prohibitively) costly ^40^, but it is to be hoped that far less costly small molecules to achieve this end will be developed before long.

### Treating arteries instead of treating risk factors

**Figure 2.**
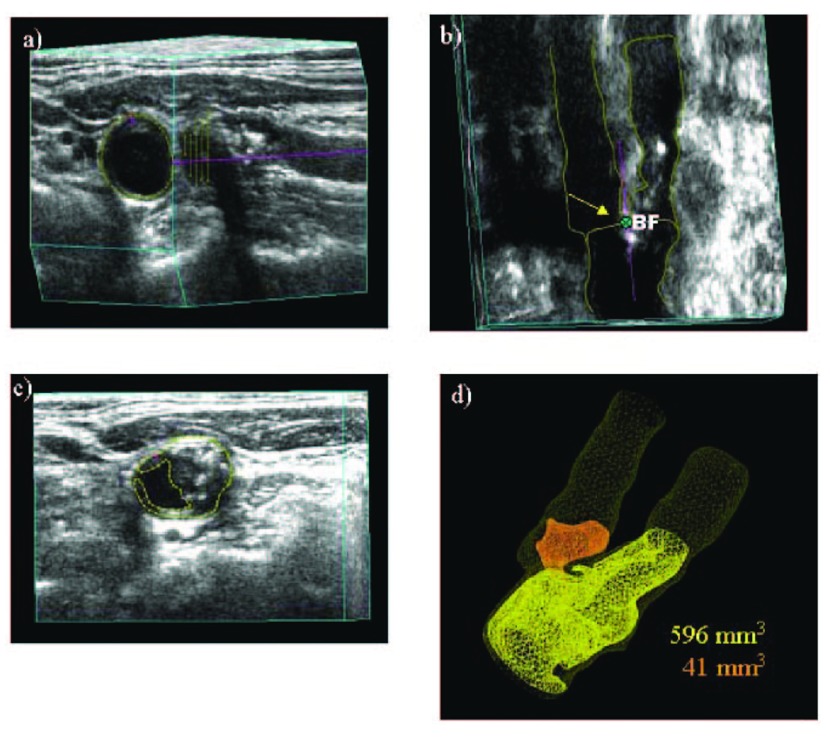
Procedure for determining plaque volumes from 3D ultrasound images. **a**) An approximate axis of the vessel is selected in a longitudinal view (colored line) and the internal elastic lamina and lumen boundary are outlined (yellow). **b**) Using the surfaces generated by the vessel contours and the 3D ultrasound image, the position of the bifurcation (BF; yellow arrow) is determined and marked. The axis of the vessel is selected based on the bifurcation point and marked along the branch as far as the plaque can be measured (colored line). This axis will be used as a reference for distance measurements. **c**) All plaques within the measurable distance are outlined, different colors being used for each separate plaque to aid in identification. **d**) Volumes are calculated for each plaque, and surfaces of the vessel wall and plaques are generated to better visualize the plaques in relation to the carotid arteries. Reproduced by permission of Wolters Kluwer from: Ainsworth CD, Blake CC, Tamayo A, Beletsky V, Fenster A, Spence JD: 3D ultrasound measurement of change in carotid plaque volume: a tool for rapid evaluation of new therapies. Stroke 2005, 36(9): 1904–1909.

A different approach to reducing residual risk is to treat the actual burden of atherosclerosis ^41^ instead of treating intermediate targets—risk factors such as level of blood pressure or LDL ^10^. This paradigm was developed in response to the recognition that treating patients according to then current guidelines was failing half our patients: they had plaque progression, and their risk was twice that of patients with stable plaque or plaque regression, after controlling for coronary risk factors ^42^. This approach, initiated by our group in 2003, was found in 2010 ^11^ to halve the proportion of patients with plaque progression (to a quarter), double the proportion of patients with regression of plaque (to half) ^10^, reduce microemboli on TCD by three quarters, and reduce the very high risk of patients with asymptomatic carotid stenosis by over 80% ^11^: the 2-year risk of stroke fell from 8.8% to 1% and the 2-year risk of myocardial infarction from 7.6% to 1%. Efforts are underway to conduct randomized trials of usual care versus “treating arteries” using measurement of 3D plaque volume ( Figure 2), the most sensitive method available to assess the effects of therapies on atherosclerosis ^43, 44^.

## Assessing prognosis in carotid atherosclerosis

Although it has been reasonably clear that patients with severe symptomatic carotid stenosis benefit from CEA or CAS, the periprocedural risk of stroke or death with CAS is approximately twice that with CEA. However, the risk of asymptomatic carotid stenosis with modern medical therapy has declined markedly in recent years to ~0.5% per year ^11, 45, 46^, so it has even been suggested ^47^ that randomized trials in *symptomatic* stenosis should be repeated comparing intervention with intensive medical therapy. There is a major unwarranted controversy regarding the treatment of asymptomatic carotid stenosis. In the United States, ~90% of carotid intervention is for asymptomatic stenosis, even though 90% of patients would be better treated with intensive medical therapy ^12^. This is being justified by an invalid extrapolation from the medical risks in randomized trials conducted decades ago to modern risks with CEA and CAS. Although the most recent trials report that the risk of stroke or death after first deducting periprocedural risk is similar to that with modern intensive medical therapy (~0.5% per year), the risk with intervention is still much higher than the risk with medical therapy once the periprocedural risks are taken into account: ~3% with CAS and 1.5% with CEA. Furthermore, there are important caveats regarding the higher risk in real-world practice as opposed to the risks with carefully vetted interventionalists in randomized trials ^48, 49^. There are huge discrepancies across the world in the proportion of carotid interventions performed for asymptomatic stenosis: 90% in the US, ~60% in Italy and Germany, ~15% in Canada and Australia (about right), and 0% in Denmark. These discrepancies call into question not only the advisability but also the ethics of routine intervention for asymptomatic stenosis, as practiced in the United States. The reasons for this practice do not bear scrutiny ^50, 51^.

Approximately 10–15% of patients with asymptomatic stenosis could benefit from intervention, and fortunately there are methods available to identify them. Reduced cerebrovascular blood flow reserve is one approach that appears to be promising. In development are a number of other approaches based on imaging of vulnerable plaque, including intraplaque hemorrhage on magnetic resonance imaging (MRI) scans, neovascularity of plaques with contrast ultrasound, and plaque inflammation on positron emission tomography/computed tomography (PET/CT) scans ^13^. Here, I will focus on the uses of ultrasound in assessing prognosis in patients with carotid atherosclerosis.

## Measuring carotid plaque burden

**Figure 3.**
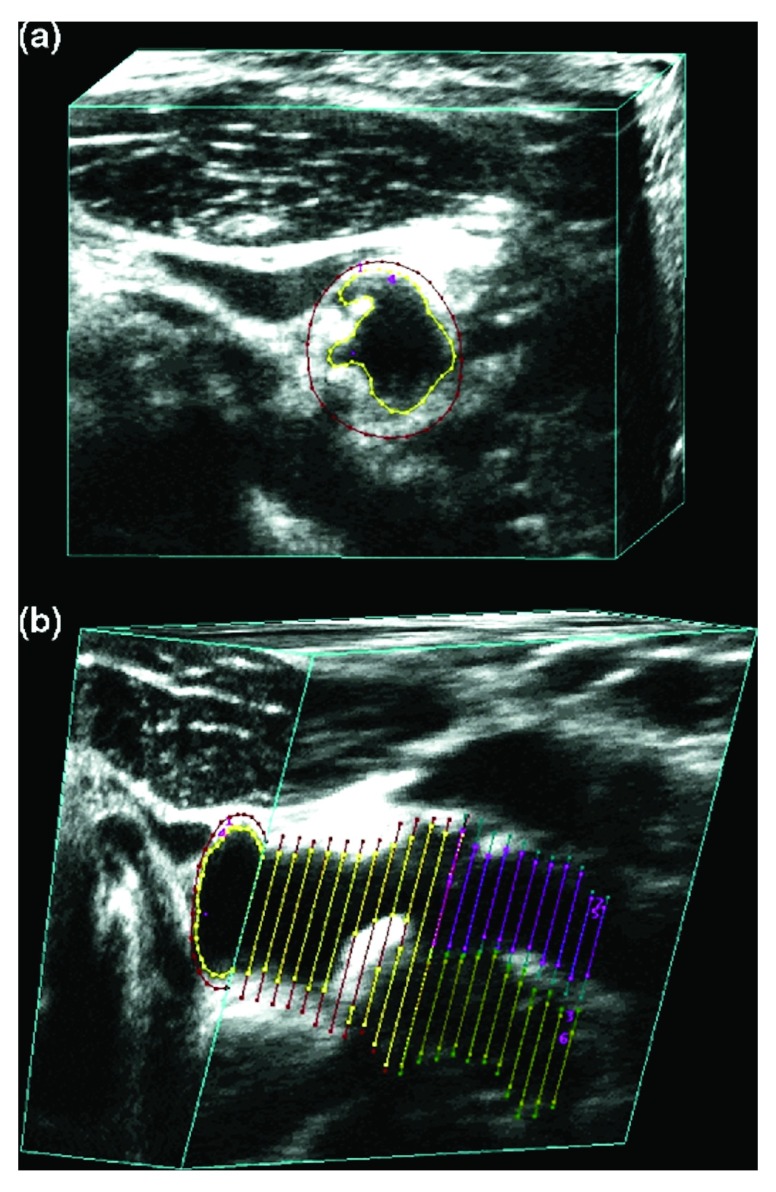
Vessel wall volume. Vessel wall volume segmentation. ( **a**) The transverse view of the common carotid artery shows the vessel boundary outlined in red and the lumen boundary outlined in yellow. ( **b**) The 3D ultrasound image volume is sliced longitudinally to reveal the vessel and lumen boundaries in the common, internal, and external carotid branches. The internal carotid artery vessel and lumen boundaries are shown in blue and pink, respectively. Reproduced by permission of Elsevier from: Egger M, Spence JD, Fenster A, Parraga G: Validation of 3d ultrasound vessel wall volume: an imaging phenotype of carotid atherosclerosis. Ultrasound Med Biol 2007, 33(6): 905–914.

Although widely regarded as “preclinical atherosclerosis”, carotid intima-media thickness (IMT) is a different phenotype. This issue is complicated by two different approaches to the measurement of IMT—with and without plaque thickness—(the latter being a one-dimensional measurement of plaque in those participants with plaque). Then in studies in which plaque thickness is included, participants with and without plaque thickness are combined, conflating the issue ^52^. It is increasingly clear that measuring carotid plaque burden is superior to measuring IMT, both for risk stratification and for assessment of effects of therapy ^53^. Plaque burden can be measured as total plaque area (TPA) ^42^ (the sum of the areas of all plaques seen in the extracranial carotid arteries) or total plaque volume (TPV) ^54^. In the High Risk Plaque study, 3D plaque burden was highly correlated with coronary calcium, whereas IMT was not ^55^, and plaque burden predicted risk of cardiovascular events to a similar extent as coronary calcium ^56^. Often plaque volume is measured not as TPV but as the volume over a defined segment of the carotids, limited to a defined distance above and below the bifurcation. This approach has advantages for such purposes as assessing effects of therapy (simplicity, potential for automatic measurement) but may lose dynamic range, a potential issue with regard to prognostic value. Besides the issue of IMT not truly representing atherosclerosis, a key reason why IMT is a weak predictor of cardiovascular risk ^57^ is its narrow dynamic range: ~0.5 to 1.5 mm. The dynamic range of TPA is much greater – from 0 to ~1200 mm ^2^; the range of TPV would be even greater. After adjustment for age, sex, blood pressure, smoking, serum cholesterol, diabetes, homocysteine, and treatment of blood pressure and cholesterol, TPA strongly predicted risk among patients attending a vascular prevention clinic: the 5-year risk of stroke, death, or myocardial infarction, by quartile of TPA, was 5.6%, 10.7%, 13.9%, and 19.5%. Plaque progression also increased risk; patients with plaque progression in the first year of follow up had twice the risk of those with stable plaque or plaque regression. These findings were substantiated in a population-based study in Tromsø, Norway ^58, 59^. Myocardial infarction and stroke were both strongly predicted by TPA but not by IMT in the common carotid where there was no plaque. The annual change in IMT is too small in relation to the resolution of the method to permit measurement in time frames that are clinically meaningful: the annual change is ~0.15 mm, and the resolution of the method is ~0.2–0.3 mm. A much more useful measurement in persons who do not have plaque would be to measure vessel wall volume ( Figure 3) ^60^; this parameter amounts to a 3D measurement of the intima-media, has a much greater dynamic range, and unlike IMT, is sensitive to effects of therapy ^61, 62^. Progression of IMT does not predict risk ^63^, even in large populations, whereas progression of 3D plaque volume predicted cardiovascular events in a small population of patients in our study ^64^.

## Transcranial Doppler embolus detection

**Figure 4.**
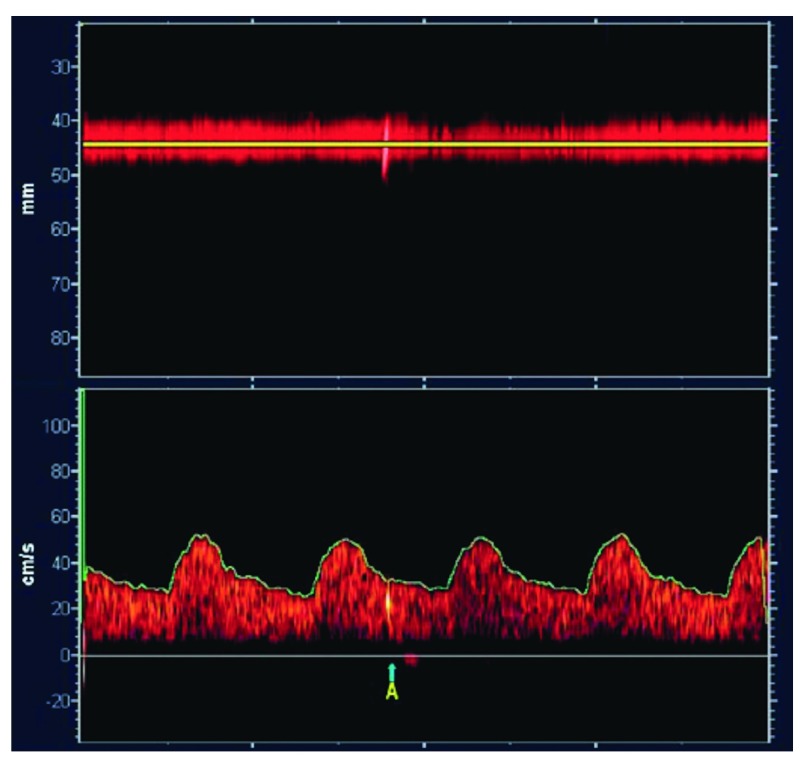
Transcranial Doppler embolus detection. Microembolus in a patient with asymptomatic carotid stenosis. The upper channel is an M-mode image of an embolus in the middle cerebral artery; the lower panel shows the high-intensity transit signal in the Doppler channel. Besides the visual appearance of the microembolus, a characteristic clicking sound is heard. Reproduced by permission of the Society for Vascular Ultrasound from: Spence JD. Transcranial Doppler: uses in stroke prevention. The Journal for Vascular Ultrasound 2015, 39(4): 183–187.

Perhaps the best validated method for identifying high-risk patients with asymptomatic carotid stenosis is TCD embolus detection ^12, 65^. Figure 4 shows a microembolus in a patient with asymptomatic stenosis. Patients with asymptomatic carotid stenosis who had two or more microemboli in 1 hour of monitoring had a 1-year risk of stroke of 15.6%, indicating that they could benefit from CEA or CAS. The cost of a TCD machine is less than the cost of two CAS, and training and certification in TCD embolus detection can be obtained in a course of 3 or fewer days. Thus TCD embolus detection or some other procedure to identify the patient as being at a higher risk than that with intervention should be considered before patients undergo CAS or CEA for asymptomatic stenosis.

## Plaque characteristics and prediction of risk

In patients with asymptomatic carotid stenosis, the presence of three or more ulcers in either or both carotid arteries carried a similar risk as the presence of microemboli: an 18% 3-year risk of stroke or death. Those with two or more microemboli had a 20% 3-year risk ^66^. By combining TCD embolus detection and detection of three or more ulcers, the proportion of patients with asymptomatic stenosis who could benefit from intervention was increased from 5% to 10%. In the Asymptomatic Carotid Emboli Study (ACES) ^67^, plaque echolucency at baseline increased the risk of ipsilateral stroke (hazard ratio [HR] 6.43, 95% confidence interval [CI] 1.36–30.44, P=0.019). A combination of plaque echolucency and presence of TCD microemboli markedly increased the risk of ipsilateral stroke (HR 10.61, 95% CI 2.98–37.82, P=0.0003). This association remained significant after controlling for risk factors, degree of carotid stenosis, and antiplatelet medication. Juxtaluminal black plaque (plaque or thrombus that is so echolucent that it can be seen only by observing a gap between the wall and the Doppler flow signal) ^68^, plaque echolucency, and plaque texture analysis of ultrasound ^69^ identified higher risk.

**Figure 5.**
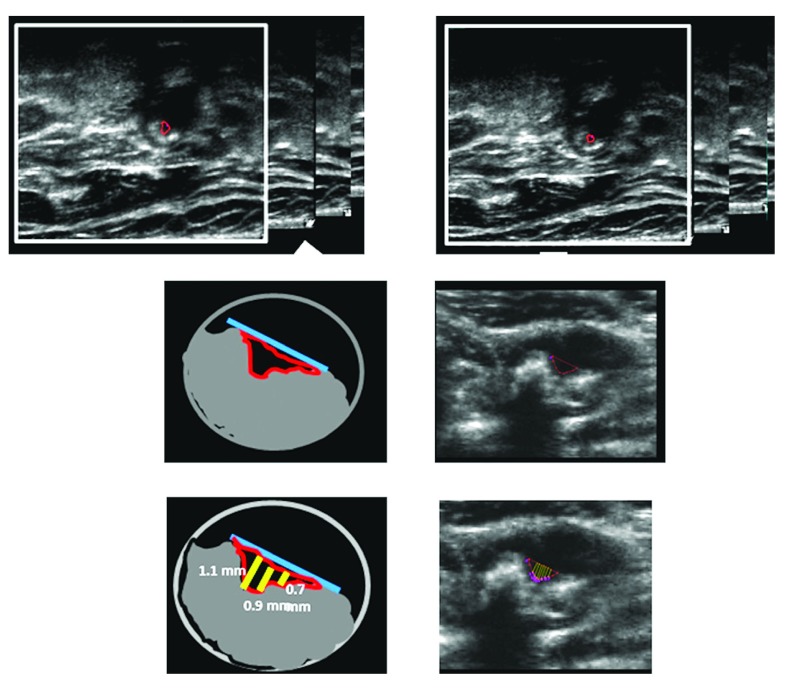
Carotid ulcer volume. Measurement of ulcer volume and ulcer depth. Contours of ulcers were traced and depth of ulcers measured in cross-sectional views. Each slice had a thickness of 1 mm; ulcer volume was computed from the sum of the volumes of all slices in which ulceration was traced. Reproduced by permission of Wolters Kluwer from: Kuk M, Wannarong T, Beletsky V, Parraga G, Fenster A, Spence JD: Volume of carotid artery ulceration as a predictor of cardiovascular events. Stroke 2014, 45(5): 1437–1441.

**Figure 6.**
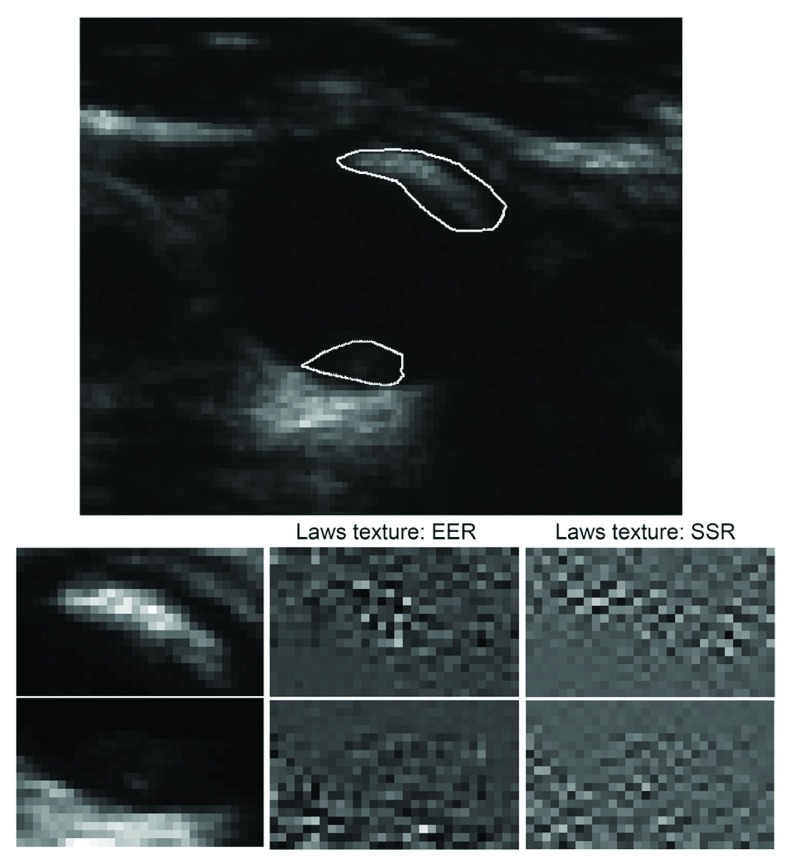
Carotid plaque texture. Texture for two plaques in the same vessel with a different appearance. In a total of 50 runs of sparse Cox regression (5× 10-fold cross-validation) on changes in texture, Laws edge-edge-ripple (EER) was selected in the model 49 times, and Laws spot-spot-ripple (SSR) 48 times. Reproduced by permission of Wolters Kluwer from: van Engelen A, Wannarong T, Parraga G, Niessen WJ, Fenster A, Spence JD, de Bruijne M: Three-dimensional carotid ultrasound plaque texture predicts vascular events. Stroke 2014, 45(9): 2695–2701.

Even in patients without carotid stenosis, plaque characteristics predict cardiovascular risk. In a relatively small sample (349) of patients attending a vascular prevention clinic, followed for 5 years, the volume of ulcers ( Figure 5) predicted risk of cardiovascular events ^70^. Patients with a total ulcer volume ≥5 mm ^3^ experienced a significantly higher risk of stroke, transient ischemic attack, or death (P=0.009) and of stroke/transient ischemic attack/death/myocardial infarction/revascularization (P=0.017). In the same patient population, elements of plaque texture identified from radiofrequency signals in ultrasound ( Figure 6) predicted cardiovascular risk ^71^.

**Figure 7.**
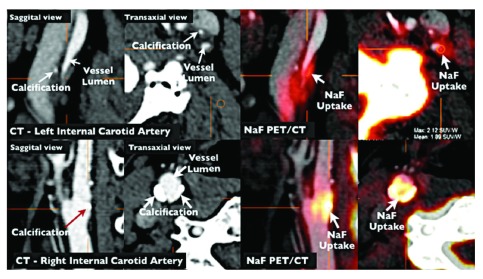
Imaging of vulnerable plaque by positron emission tomography/computed tomography (PET/CT). NaF PET/CT imaging of left and right internal carotid arteries of active calcification in a 72-year-old symptomatic patient evaluated at the University of Ottawa Heart Institute. Upper row: evidence of NaF uptake with small foci of calcification on CT in the left internal carotid symptomatic culprit vessel. There is a mismatch between the region of NaF uptake and calcification on CT. Lower row: evidence of calcium nodules with matched NaF uptake at the right internal carotid artery. Reproduced by permission of Springer from: Cocker MS, Mc Ardle AB, Spence JD, Lum C, Hammond RR, Ongaro DC, McDonald MA, Dekemp RA, Tardif JC, Beanlands RS: Imaging atherosclerosis with hybrid [18F]fluorodeoxyglucose positron emission tomography/computed tomography imaging: what Leonardo da Vinci could not see. J Nucl Cardiol 2012, 19(6): 1211–1225.

Intraplaque hemorrhage on MRI ^72– 74^ appears promising as a method for identifying high-risk asymptomatic stenosis, as does imaging of plaque inflammation on PET/CT with fluorodeoxyglucose ^75^ and imaging of active calcification with sodium fluoride ^76^ ( Figure 7). It seems likely that by combining several imaging modalities, the proportion of patients with asymptomatic stenosis who could be identified as being at high enough risk to warrant intervention might be increased to ~15%. Just as it is inappropriate to perform CAS or CEA in all patients with asymptomatic stenosis, it is also inappropriate to intervene in none: ~10% of strokes are from carotid stenosis, and most patients were asymptomatic before the event. Modalities to identify the few who could benefit from intervention should be in more widespread use; indeed no patient with asymptomatic stenosis should be subjected to intervention without first being identified as being at a high enough risk of ipsilateral stroke to benefit from intervention.

## Conclusion

Atherosclerosis is a complex process. Diet is much more important than most physicians suppose, and the intestinal microbiome has major effects on metabolic products derived from dietary constituents such as carnitine from meat and phosphatidylcholine from egg yolk. Avoidance of red meat and egg yolk is particularly important in patients with impaired renal function. New approaches to lowering LDL by blocking the effect of PCSK9, and a strategy of treating atherosclerosis directly instead of focusing on intermediate targets, show promise of reducing the residual risk that remains after current therapy. Most patients with asymptomatic carotid stenosis would be better treated with intensive medical therapy than with CEA or CAS. Microemboli on TCD, impaired cerebral blood flow reserve, juxtaluminal black plaque, echolucency, intraplaque hemorrhage on MRI, and ulceration on 3D ultrasound are ways to identify the 10–15% of patients with asymptomatic carotid stenosis who are at sufficiently high risk to benefit from intervention. Other approaches such as plaque texture on ultrasound and PET/CT imaging of inflamed plaque and early calcification will in future provide further evidence of their contribution to identifying high-risk asymptomatic carotid stenosis.

## Abbreviations

CAS, carotid artery stenting; CEA, carotid endarterectomy; CI, confidence interval; HMG CoA, hydroxymethylglutarate CoA; HR, hazard ratio; IMT, intima-media thickness; LDL, low-density lipoprotein; MRI, magnetic resonance imaging; PCSK9, proprotein convertase subtilisin-kexin type 9; PET/CT, positron emission tomography/computed tomography; TCD, transcranial Doppler; TMAO, trimethylamine N-oxide; TPA, total plaque area; TPV, total plaque volume.
